# Task-Specific Codes for Face Recognition: How they Shape the Neural Representation of Features for Detection and Individuation

**DOI:** 10.1371/journal.pone.0003978

**Published:** 2008-12-29

**Authors:** Adrian Nestor, Jean M. Vettel, Michael J. Tarr

**Affiliations:** Department of Cognitive and Linguistic Sciences, Brown University, Providence, Rhode Island, United States of America; University of Southern California, United States of America

## Abstract

**Background:**

The variety of ways in which faces are categorized makes face recognition challenging for both synthetic and biological vision systems. Here we focus on two face processing tasks, detection and individuation, and explore whether differences in task demands lead to differences both in the features most effective for automatic recognition and in the featural codes recruited by neural processing.

**Methodology/Principal Findings:**

Our study appeals to a computational framework characterizing the features representing object categories as sets of overlapping image fragments. Within this framework, we assess the extent to which task-relevant information differs across image fragments. Based on objective differences we find among task-specific representations, we test the sensitivity of the human visual system to these different face descriptions independently of one another. Both behavior and functional magnetic resonance imaging reveal effects elicited by objective task-specific levels of information. Behaviorally, recognition performance with image fragments improves with increasing task-specific information carried by different face fragments. Neurally, this sensitivity to the two tasks manifests as differential localization of neural responses across the ventral visual pathway. Fragments diagnostic for detection evoke larger neural responses than non-diagnostic ones in the right posterior fusiform gyrus and bilaterally in the inferior occipital gyrus. In contrast, fragments diagnostic for individuation evoke larger responses than non-diagnostic ones in the anterior inferior temporal gyrus. Finally, for individuation only, pattern analysis reveals sensitivity to task-specific information within the right “fusiform face area”.

**Conclusions/Significance:**

Our results demonstrate: 1) information diagnostic for face detection and individuation is roughly separable; 2) the human visual system is independently sensitive to both types of information; 3) neural responses differ according to the type of task-relevant information considered. More generally, these findings provide evidence for the computational utility and the neural validity of fragment-based visual representation and recognition.

## Introduction

One of the hallmarks of human face processing is our ability to recognize faces across a multitude of levels. At the most general level, we are able to locate and distinguish faces from non-faces in a visual scene. We can also categorize faces by any number of traits including gender, ethnicity, expression, and age. Finally, we routinely discriminate faces from one another at the individual level (which we refer to as “individuation”). This range of abilities leads us to ask whether the visual system carries out such categorization tasks using a single general type of category representation or, alternatively, translates task-specific constraints into different representations of the overall category. Our study focuses on the two ends of this categorization spectrum by comparing the sensitivity of the human visual system to face detection and face individuation.

Artificial systems for automatic face recognition typically treat detection and individuation as two separate problems with two different goals [1]. However it is less clear to what extent biological systems such as the human visual system adopt a similar dual approach. Models of human face processing acknowledge the difference between multiple face recognition tasks. For instance, two classical models of face processing [2] and its neural basis [3] are centered around task differences. However, the main dichotomy these models emphasize is that between expression recognition and individuation. While an early stage of facial feature processing is separated from these tasks, the locus of face detection as well as its relationship with this early stage and the other tasks are less spelled out. Neuroimaging results also provide mixed evidence for the neural separability of detection and individuation. Some studies found that a set of common areas in the fusiform gyrus [4]–[7] and the inferior occipital gyrus [5], [7] are sensitive to both face detection and individuation. However, a recent study [8] uncovered an area sensitive to face individuation in the right anterior inferior temporal cortex, outside the typical face-selective regions recruited by detection. Consistent with this result, studies of white matter connectivity linked behavioral individuation performance with the structural integrity of fibers connecting the fusiform gyrus with more anterior areas in the right hemisphere, including the inferior temporal cortex [9], [10]. Finally, the neural markers of the time course for these two processes also seem to be different: detection and individuation were associated with separate M100 and M170 components in a magnetoencelography study [11].

If face detection and individuation do recruit different brain areas and exhibit different time courses, this may point to processing and representational differences that characterize and motivate their separation. One possibility is that the two tasks we consider here pose objectively different constraints on the featural codes underlying these two types of face recognition. The present study investigates the neural separability of detection and individuation precisely by exploring this issue. We hypothesize that detection and individuation require separate sets of facial features to optimally achieve their goals and that the visual system adopts this separation to perform different aspects of face recognition more effectively. Moreover, we surmise this difference in the representational bases of the two tasks leads in turn to differences in neural processing sufficiently robust to be tested by functional magnetic resonance imaging (fMRI).

The present study investigates this hypothesis by appealing to a framework for synthetic vision initially developed in the context of automatic object detection [12]. More recently this framework has been extended to a model of human vision and has been evaluated with respect to its original use as a method for category detection [13], [14] and category learning [15]. Within this framework, categories of objects are represented as sets of overlapping rectangular image fragments of different aspect ratios, sizes and resolutions. Many other candidates for the role of object and facial features have been proposed in the literature: edge structures [16], [17], principal components of images [18], [19] or image segments [20], [21], to name just a few. However, for the goals of our present study, we find fragment features appealing for a number of reasons. First, they are cue-agnostic in that they do not commit themselves from the start to a single type of cue, for example, edges. Second, they naturally account for configural information, an important aspect of face processing [22], in that allowing features to overlap constrains the spatial relationship of otherwise disjoint image fragments. Finally, and most relevant for the objectives of our present study, Ullman et al's framework provides a principled means for establishing optimal *task-specific* sets of fragment features for a given category. In the case of faces, it has been shown, for instance, that such features lend themselves naturally to deal not only with detection [12] but also with other categorization tasks, for example, individuation or expression recognition [23].

We note the problem of mapping diagnostic areas for specific object categories and particular tasks has been investigated in human observers using other approaches. For instance, reverse correlation methods [24]–[26] and ‘bubbles’ [27], [28] in particular, can effectively produce maps of task-diagnostic regions of images with respect to a visually-homogeneous category such as faces. Critically, the task-diagnostic maps produced by these methods are compatible with different models of how one might divide an object into features. The fragment-based approach we adopt here produces concrete ways to decompose a stimulus category into feature components. We take advantage of this property to gain a finer-grained view of the representational codes used in task-specific face processing.

Our investigation proceeds in three stages. First, we evaluate and compare systematically the task-specific information of face fragments for detection and individuation. This evaluation enables the selection of sets of fragments whose task-specific information varies independently in the two tasks. Second, using these fragments, we assess and confirm the sensitivity of human subjects to task-diagnostic fragment features. Third and finally, an fMRI study tests and reveals that different cortical areas exhibit different patterns of sensitivity to task-specific information with respect to our two tasks. These results jointly confirm the separability of detection and individuation in the human visual system and provide evidence for different representational codes underlying the two tasks and driving the noted separation.

## Materials and Methods

### Evaluation of Task-Specific Information

#### Stimuli

Image face fragments were extracted from a set of 60 face images (12 individuals×5 expressions)–Figure S1–selected from the Tarrlab face database (available online at www.face-place.org). This set contains near-front-view grayscale Caucasian faces, half of which were male and half female, wearing no glasses or other facial accessories and displaying variable affective expressions. The faces were cropped, down-sampled (60×40 pixels) and normalized with the position of the main features, the eyes and the nose, to permit the mapping of corresponding face fragments across faces. A similar set containing 12 different individuals was used for cross-validation of the computational results. In addition, two sets of 605 natural scene images were randomly selected from the McGill Calibrated Color Image Database (tabby.vision.mcgill.ca). These images, mapped to grayscale, were used in the computation of the amount of detection-specific information and also provided non-face stimuli for behavioral testing.

**Figure 1:**
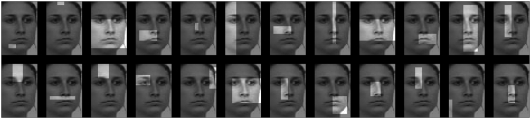
Example of face fragments extracted from the same face (displayed in reduced contrast).

The image fragments were rectangular image patches of different sizes and aspect ratios [12]. More precisely, we systematically varied the size, aspect ratio and position of a rectangular window across each face in steps of 4 pixels. Thus, the smallest fragments were 4×4 pixel image patches while the largest ones contained an entire face. For each face this procedure yielded 6600 image fragments. Consequently, application of the same procedure to all face images yielded 6600 *fragment types*, where by fragment type we mean a class of fragments corresponding to the same area of the face across different images of the same or different individuals. Examples of face fragments extracted from the same image are shown in Figure 1.

#### Methods

The amount of face-detection information was computed for each of 396000 fragments generated by extracting 6600 rectangular fragment types from 60 face images. We refer to these fragments as well as the face images from which they were extracted as the training set. A separate test set was composed of an equal number of fragments extracted from a different set of face images.

To estimate the task-specific information of a fragment of a given type *k*, we cross-correlated the given fragment with all face and non-face images. If the maximum correlation value surpassed a certain threshold θ_k_, the fragment was considered present in the image. The threshold θ_k_ was computed for each fragment type *k* so as to maximize the average task-specific information of the fragments of this type for face detection. Computation of the amount of task-specific information carried by each fragment was implemented following the original description of the method [12]. Briefly, for each face fragment we computed these values using the mutual information between fragment presence and image category, that is, face or non-face. The mutual information was computed as [29]:
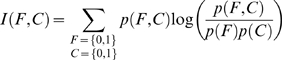



Here F is a binary variable indicating whether a given fragment was present in an image or not and C is a binary variable indicating whether the image contains a face or not. The threshold θ_k_ was estimated by maximizing the mutual information of a fragment over the training set (the best threshold was found by brute-force search from −0.99 to 0.99 in steps of 0.01). In a departure from the original method, a common threshold was estimated for each fragment type rather than for each fragment separately. The overall task-specific information for a fragment type was computed as the average mutual information of all fragments of that type in the training set.

The method described above was next extended to individuation. For this task the training set was limited to faces and C denotes in this case same/different individuals instead of face/non-face information. More precisely, C encodes whether an image contains the face of the same individual from which the fragment tested was extracted or not–for any particular fragment there are 4 different images of the same individual in addition to the one from which the fragment was initially extracted and another 55 faces of 11 different individuals. A new set of task-specific thresholds for fragment presence was estimated again for each fragment type.

We note that a given face fragment can fail to be informative for detection because it is not similar enough to other fragments of the same type, because it is highly similar to recurrent image structures found in non-face images or because of both. (Figure S2 shows natural image fragments erroneously labeled as face fragments by the method due to their similarity to actual face fragments.) It is possible a fragment is highly diagnostic of a particular individual but, due to the high variability of the type to which it belongs across different individuals, it is less useful for detection. Thus, in order to be diagnostic for the two tasks, fragment types need to satisfy two different criteria: small *extrapersonal* (between-individual) variability for detection versus large *extrapersonal* variability relative to *intrapersonal* (within-individual) variability for individuation [30]. If the two criteria conflict for most fragments, we would expect a relatively low correlation between their task-specific information values for the two tasks. To verify this hypothesis, we computed the Pearson correlation between the mutual information for detection and individuation across all fragment types.

For cross-validation purposes, task-specific information for each fragment type as well as the correlation between values for detection-specific and individuation-specific information were computed again within the test set. Cross-correlation thresholds in this case were kept fixed at the values that maximized task-specific information within the training set.

### Behavioral Experiment 1–Face Detection

#### Participants

Sixteen adults from the Brown University community volunteered to participate in the experiment in exchange for pay. All participants had normal or corrected-to-normal vision. All participants provided written consents and procedures were approved by the Institutional Review Board of Brown University.

#### Stimuli

From our 6600 face fragment types, we preselected a subset adequate for the testing of human participants. For every fragment type we verified whether it contained any subfragments with the value of detection-specific information higher or equal to that of its own. If this was not the case, the fragment type in question was included in the mentioned subset. The fact that the overall task-specific information for a fragment can be lower than that of a subfragment it contains owes to the fact that the evaluation of its task-specific information weighs in equally all pixels. The selection criterion imposed above ensures the stimuli used are categorized correctly with respect to the amount of information they provide to our observers. In its absence, overall task-specific information may be misleading in studying human vision in that participants can zero in on the most diagnostic subfragment(s) of a given fragment and disregard the rest.

Next, forty fragment types were selected for each of three levels of detection-specific information (high, middle and low) from our preselected set of candidates. Fragment types were selected so as to homogenize the entire set of stimuli with respect to potential confounds. Task-specific information for the irrelevant task, that is, individuation, as well as geometric properties, size (in number of pixels) and aspect ratio, were all considered in selecting the final set of stimuli (*p*>0.1 for all pairwise comparisons between the different levels of task-specific information across irrelevant dimensions). Finally, for each of the forty fragment types, the actual stimuli were selected by picking two fragments of that type from two randomly selected faces of two different individuals. In addition, 240 natural image fragments were extracted to match the face fragments with respect to their geometric properties. Contrast and mean luminance was equalized across all face and natural image fragments.

We note that the qualitative labels applied to the three levels of task-specific information are meaningful relative to each other rather than by absolute ranking with respect to ideally diagnostic fragments–see Table 1. This is not a reason of concern for recognition performance in that recognition, in this case detection, does not rely on single ideal features but on multiple features that jointly contribute to the process possibly based on their independent amounts of information they provide [12].

**Table 1:** Task-specific information* carried by fragments used in the two behavioral tasks.

Level of Information	Detection task		Individuation task	
	detection MI	individuation MI	detection MI	individuation MI
high	0.79 (0.02)	0.28 (0.03)	0.70 (0.09)	0.75 (0.03)
middle	0.51 (0.05)	0.25 (0.06)	0.65 (0.11)	0.55 (0.03)
low	0.21 (0.02)	0.3 (0.11)	0.64 (0.9)	0.25 (0.03)

* task-specific information is presented here as the average mutual information (MI) and the standard deviation of each fragment set relative to the mutual information of a task-ideal fragment (one that is detected in all and only those instances in which the class of interest is present).

#### Task

Participants were asked to perform a face detection task by pressing one of two buttons on a buttonbox. More precisely, participants were asked to judge whether the single image fragment displayed at a time was a face fragment or not. The response was made by pushing one of two buttons with the index fingers of the left and right hands randomly assigned to signal a face/non-face response across participants.

On each trial, a cross was presented in the center of the screen for 400 ms followed by an image fragment for 250 ms. A black screen replaced the stimulus until the participant made a response signaling the end of a trial. A stimulus subtended on the average a visual angle of 2.1×2.3 from a distance of 70 cm after doubling the size of the image by pixel replication. Each participant completed 480 trials over the course of two blocks in a 30-minute session. Each stimulus was shown only once in the entire experimental session. Trial order was randomized for each participant.

Experimental trials were preceded by a short practice session allowing the participants to familiarize with the task and the stimuli. At the end of the experiment participants were asked to report whether they were familiar with and able to recognize any of the individuals whose faces were presented in the experiment.

Stimulus design and presentation relied on Matlab 7.5 (Mathworks, Natick, MA, USA) and the Psychophysics Toolbox 3 [31], [32] running on an Apple Macintosh using OS X.

### Behavioral Experiment 2–Face Individuation

#### Participants

Another sixteen adults from the Brown University community with normal or corrected-to-normal vision participated in the experiment. All participants provided written consents and procedures were approved by the Institutional Review Board of Brown University.

#### Stimuli

From all our face fragment types, we preselected a subset in a manner analogous to the one described for the first experiment. However, this time the relevant task-specific information was computed for individuation instead of detection.

Next, the procedure described above was followed to select 40 fragment types for each of three levels of individuation-specific information. In addition to controlling for low-level properties of the stimuli we also attempted to homogenize the overall set with respect to detection-specific information–see Table 1. Finally, for each of the forty fragment types, the actual stimuli were selected by picking four fragments of that type from two individuals where each of these individuals supplied two distinct fragments of that type showing two different expressions.

Interestingly, we note that, as a result of our selection procedure, the average size of a fragment in this experiment was significantly larger than the one in the first experiment (two-tailed *t*-test *p*<0.01). This difference is mainly due to the fact that it is more difficult to find intermediate-sized fragments with small detection-specific information than it is to find small fragments with high task-specific information. Conversely, it is more difficult to find small fragments with high individuation-specific information than to find intermediate-sized fragments with low amounts of relevant information.

#### Task

Participants were asked to judge whether two fragments *of the same type* shown in succession belonged to the same individual or not. Each trial had the following structure: a cross appeared for 400 ms in the center of the screen followed by the first image fragment for 250 ms, a white noise mask with the same size as the previously presented image for 200 ms, the second face fragment for another 250 ms and a black screen until the subject made a button press. Trials were equally divided between same-individual versus different–individual trials for each condition. A stimulus subtended on the average a visual angle of 3.4×2.3 from a distance of 70 cm after doubling the size of the image. Each participant completed 240 trials over the course of two blocks in a single a 45-minute session. Each stimulus was shown only once in the entire experimental session and trial order was randomized for each participant. In all other respects we followed the procedure described for Experiment 1.

### Functional Magnetic Resonance Imaging (fMRI) Experiment

#### Participants

Eleven healthy adult members (7 female, age range: 18–30) of the Brown University community, including one of the authors JMV, volunteered to participate in the experiment for pay. None of them took part in the behavioral experiments described above. Participants were right-handed, with normal or corrected-to-normal vision and reported no contraindications for MRI scanning. All participants provided written consents and procedures were approved by the Institutional Review Board of Brown University.

#### Stimuli and behavioral task

Participants were presented with the same face fragment stimuli as the ones from the behavioral experiments but using only two levels of task-specific information: high and low. Participants lay supine and viewed the rear-projection display through an angled mirror in the bore of the magnet. Stimuli were presented in 30-second blocks of face fragments with high/low levels of information for detection or individuation. The order of the blocks was counterbalanced across participants. Half of the stimuli in each condition were presented twice during the experiment but at most once within a block. Stimulus duration was 800 ms with 700 ms inter-stimulus interval (20 stimuli per block). The stimuli in the detection and individuation conditions subtended different viewing angles similar to those in the behavioral experiment. In each time-series there were four stimulus blocks separated by 30-second fixation blocks during which participants were instructed to look at a fixation cross displayed in the center of the screen. In total, we acquired 6 time series with image fragments and 2 additional time series with blocks of faces and objects serving as a standard face-localizer.

On every trial the participants performed an unrelated task. The stimuli were randomly jittered 1° to the left/right of the center fixation cross and the participants performed a one-back location task by pushing one of two buttons randomly assigned with the index fingers of the two hands.

#### Scanning parameters

Scanning was carried out at the Brown University MRI Research Facility with a Siemens 3T TIM Trio magnet with an 8-channel phased-array head coil. Functional images were acquired with an ascending interleaved echo-planar imaging (EPI) pulse sequence (90 time points per time series; TR = 3 s; TE = 30 ms; flip angle 90°; 3 mm isotropic voxels; field of view 192×192×144 mm^3^; 48 slices covering the entire cerebral cortex). At the beginning of each session, we also acquired a T1-weighted anatomical image (1 mm isotropic voxels; 160 slices of size 256×256 mm).

#### Analysis of imaging data

Analysis was carried out using AFNI [33] and custom in-house Matlab (Mathworks, Natick MA) code. The first 5 images of each fMRI time series, during which subjects maintained fixation, were removed to allow the hemodynamics to achieve a steady state and to minimize transient effects of magnetic saturation. Further preprocessing involved slice scan time correction, 3-D motion correction, smoothing with a Gaussian kernel of 6 mm FWHM, normalization (each voxels's time series was divided by its mean intensity to convert the data from arbitrary image intensity units to percent signal modulation) and linear trend removal. Group analyses were performed after converting functional images into Talairach space [34].

Conventional univariate mapping analysis was performed on each participant's data. For each experimental condition we constructed a box-car predictor and convolved it with a gamma function. The general linear model [35] was applied to compute the coefficient of each predictor independently for each voxel. Significance maps of the brain were computed by t-tests of pairwise comparisons between relevant conditions. Significance levels were corrected by taking into account cluster size and its false detection probability[36] (*p* = 0.05 corrected). This type of analysis was used to contrast the high and low information conditions for detection and individuation as well as faces versus objects in a standard face localizer test [7], [37].

In addition, we performed multivariate pattern analysis [38] to distinguish between the two levels of task-specific information for each task in face selective areas. Principal component analysis (PCA) was first applied to the coefficients of all voxels in a given area across all blocks to reduce the dimensionality of the patterns. A ‘leave-one-out’ (‘jackknife’) classification procedure was then carried out on the resultant patterns. More precisely, we trained a linear classifier on the patterns corresponding to all blocks except one and tested it on this remaining pattern. This procedure was repeated in turn for all blocks, every time leaving out a different pattern. For the purposes of pattern classification we used a linear support vector machine (SVM)–other classifiers we tested, such as a single-layer perceptron, yielded similar results. Importantly, multivariate analysis was carried out on a version of the data that had not been spatially smoothed, thus preserving high-frequency spatial information.

## Results

### Task-Specific Fragment Information for Detection and Individuation

**Figure 2:**
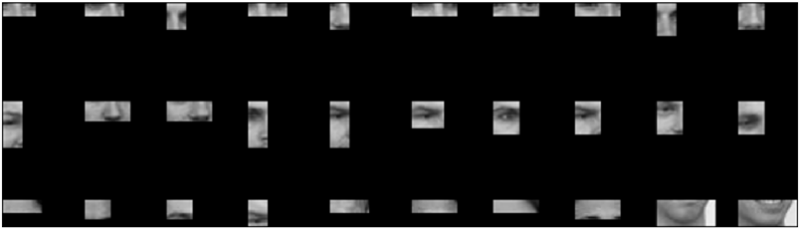
Face fragments with high (top), intermediate (middle) or low (bottom) levels of detection-specific information.

**Figure 3:**
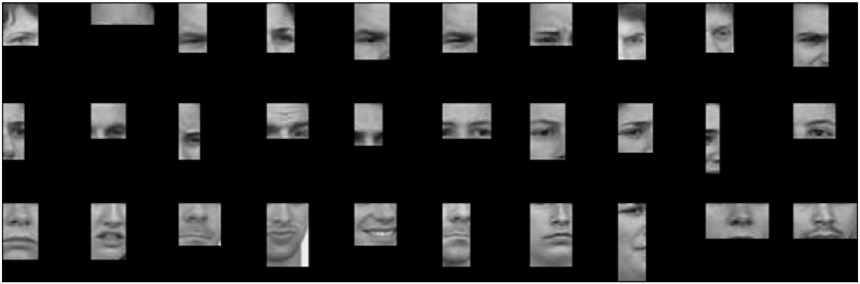
Face fragments with high (top), intermediate (middle) or low (bottom) individuation-specific information.

Task-specific information values for fragment types reliably transferred from the training face dataset to a new dataset. The correlation between the scores for the training and the test dataset were significant for both detection (r = 0.93, *p*<0.001) and individuation (r = 0.92, *p*<0.001). Fragments at three levels of information, high, middle and low,–see Table 1–are shown in Figure 2 for detection and in Figure 3 for individuation. While fragments at each level of information covered together most of the face, we note several tendencies. In the case of detection, the most diagnostic fragments tended to span the area between the eyes, less diagnostic ones tended to contain only one feature such as one eye or the nose and the least diagnostic ones contained the hairline or the chin. For individuation, highly and intermediately diagnostic fragments contained the top part of the face while the least informative ones contained the lower part of the face, the chin, the mouth and the lower nose.

As far as the relationship between the two types of task-specific information is concerned, the comparison of the scores for detection and individuation showed a weak albeit significant correlation both within the training set (r = 0.25, *p*<0.001) and within the test set (r = 0.23, *p*<0.001). These results suggest that the two types of information may be roughly separable from one another. In line with this suggestion and providing further confirmation for it, we were able to manipulate one type of task-specific information independently of the other when selecting our experimental stimuli while controlling at the same time for low-level properties of the fragments.

### Behavioral Results–Experiments 1 and 2

None of the participants were able to correctly identify any individuals familiar to them from experience prior to the experiments.

Accuracy scores for each participant were computed using d', a signal detection measure of discrimination performance between two classes combining the relative contribution of hits and false alarms [39]. In the case of detection, hits and false alarms were provided by correct and incorrect ‘face’ responses, while for individuation they were provided by correct and incorrect ‘same-individual’ responses.

**Figure 4:**
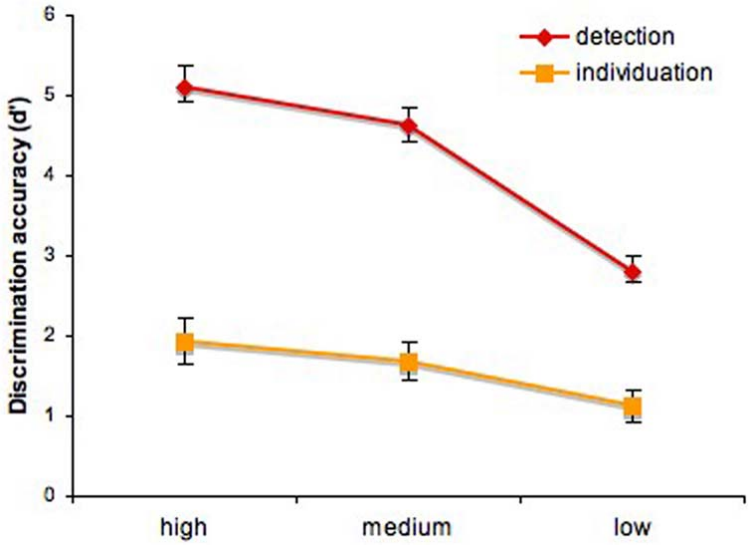
Discrimination accuracy for detection and individuation across three levels of task-specific information (mean±SEM).

Repeated measures analysis of variance was conducted across the discrimination performance of the participants in each experiment. We found a main effect of information level for both detection (*F*(15, 30) = 3.92, *p*<0.001) and individuation (*F*(15, 30) = 4.88, *p*<0.001) indicating that participants are less accurate at recognizing faces with decreasing amounts of relevant information–Figure 4. In addition, we performed a two-way analysis of variance across the level of information and task combining the results of the two experiments. The analysis revealed significant effects for both the task factor (*F*(1, 90) = 231.71, *p*<0.001) and the interaction of the two factors (*F*(2, 90) = 7.33, *p*<0.01). We also note that performance was above chance for all information levels in both experiments (significantly above d' = 0).

**Figure 5:**
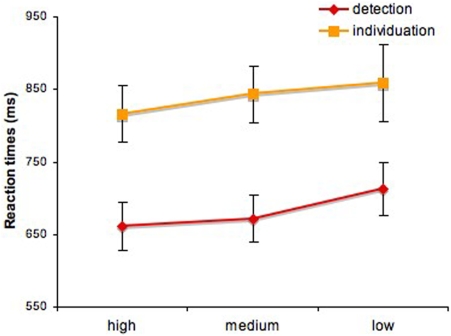
Reaction times for detection and individuation across three levels of task-specific information (mean±SEM).

Similar analyses computed for reaction times revealed no significant effect of task-specific information for either task (*p*>0.1)–Figure 5. In the two-way analysis of variance, we found a main effect of task (*F*(1, 90) = 23.63, *p*<0.001) but no significant interaction (*p*>0.5). The main effect of the task is a good indicator of task difficulty: face individuation was more difficult than face detection despite the larger size of the stimuli as reflected by both discrimination performance and reaction times.

### fMRI Results

**Figure 6:**
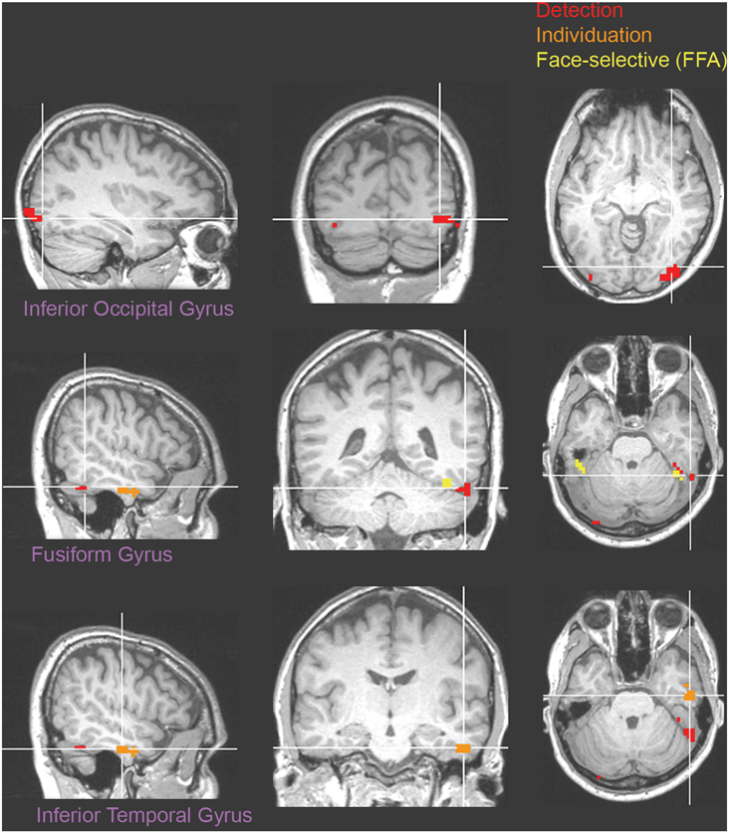
Group map superimposed on the brain of one participant.

Group maps of task-specific information effects were obtained by averaging the statistical parametric maps of individual participants–Figure 6. The comparison of the two detection conditions revealed two areas more active for diagnostic fragments than non-diagnostic fragments across participants (*p*<0.05, corrected): a region in the right posterior fusiform gyrus (pFG) and a bilateral region in the inferior occipital gyrus (IOG)–see Table 2. The first of these regions borders the functionally-localized face-selective region we find in the right fusiform gyrus, also known as the right ‘fusiform face area’ (FFA) [27], while the second surrounds and completely contains the functionally-localized face-selective region we found in the right IOG, the right ‘occipital face area’ (OFA) [26]. (Consistent with other studies, the left OFA was not reliably found across a number of participants and therefore excluded from analyses). In contrast, the comparison of the two individuation conditions revealed one area more active in the right anterior inferior temporal gyrus (aIT).

**Table 2:** Areas sensitive to task-specific information.

Task	Region	Coordinates (center)			Size (mm^3^)	Peak *t*-value
		x	y	z		
detection	R.IOG	33	−86	−6	1647	6.98
detection	L.IOG	−36	−83	−10	648	5.64
detection	R.FG	48	−45	−25	621	4.50
individuation	R.aIT	50	−9	−28	702	4.33

**Figure 7:**
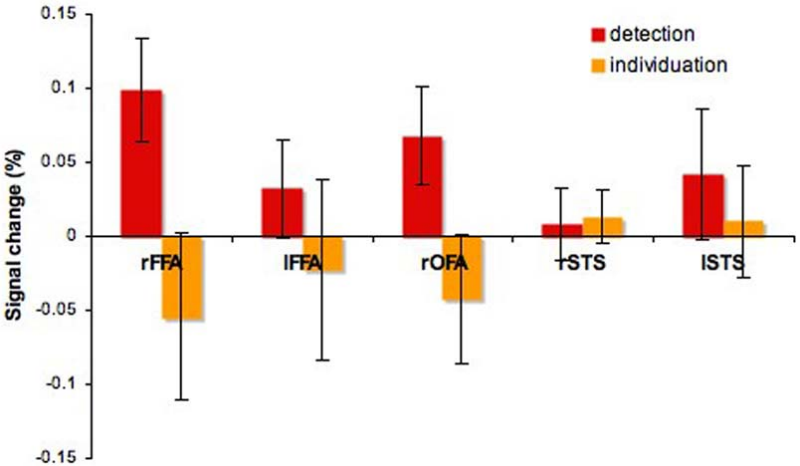
Task-specific information effects in face-selective regions (mean±SEM).

Specific region-of-interest analyses were performed in the functionally-localized face-selective areas nominally forming the core system for face processing [3]: the FFA, the OFA and another region located bilaterally in the superior temporal sulcus (STS). The areas were individually localized across participants using the data from the standard face-localizer scans, and the average percentage signal change was computed for each area and each task. Detection effects were reliably found in the right FFA (*t*(7) = 4.14, p<0.05) and the right OFA (*t*(7) = 3.22, p<0.05)–Figure 7. In contrast, no individuation-specific information effects were found in any of these regions (*p*>0.1).

In addition to the analysis of face-selective regions of interest, we examined the response of regions localized for one task, with the other task as well; for example, we examined whether there is sensitivity to detection-specific information in the aIT region already identified as sensitive to individuation-specific information. No significant effects were found for any of these comparisons.

**Figure 8:**
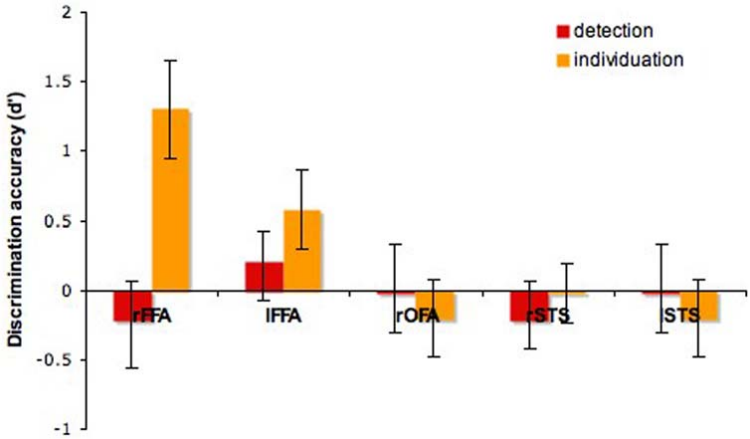
Pattern discrimination performance for the two tasks in face-selective regions (mean±SEM).

Next, pattern analysis was applied across blocks within each face-selective region for each subject. The discriminability of the two levels of information for each task was encoded using again the d' measure. Neural responses elicited by higher-information fragments were encoded as hits or false alarms when recognized correctly and incorrectly, respectively. The only region that showed significant sensitivity across participants was the right FFA when viewing fragments varying across levels of individuation-specific information (*t*(7) = 3.67, p<0.01)–Figure 8. No other region was significantly different from chance for either task (*p*>0.05).

## Discussion

Our study examines how different tasks impact the featural code used in human face processing. From the many tasks that constitute face recognition, we focused on detection and individuation in that they represent two ends of the face recognition spectrum. This comparison is made tractable by adopting a general computational framework–fragment-based category representations. The concrete question we ask within this framework is twofold: how does the task-specific information carried by face fragments objectively vary within and across tasks and how sensitive is the visual system to these types of variation?

First, from a computational perspective, we find that the two types of task-specific information, for detection and individuation, are roughly separable when considering the mutual information between fragment presence and the category of interest. This result is not entirely unexpected given that in order to be diagnostic for the two tasks a fragment type would need to satisfy two criteria presumably at tension with each other. For detection, the area of the face captured by a fragment would need to exhibit small *extrapersonal* variability and be visually dissimilar from recurrent non-face image structures. For individuation, on the other hand, the same area would need to exhibit large *extrapersonal* variability relative to *intrapersonal* variability [30]. Consistent with this tension, the correlation we found between the two types of task-specific information is relatively small although still significant. The small size of this correlation is what justifies and explains our ability to select (with relative ease) two subsets of fragments that vary independently with respect to their task-specific information for the two tasks. We then use these separate subsets to examine the relationship between detection and identification in human visual processing–a high correlation would have made this analysis less likely to succeed. To be clear, we are not using this small, but significant correlation to argue alone for the separability of detection and individuation features, nor are we arguing for complete separability. Instead we suggest that partial but reliable separability occurs with regard to task-specific features. Based on these results, it would appear, detection-diagnostic fragments should still be able to support individuation, albeit in a non-optimal fashion, and vice versa. The extent to which this prediction holds for automatic recognition should be the subject of further investigation. Interestingly, our neuroimaging results hint that this may indeed be the case with face processing in the human visual system.

Second, our behavioral and neuroimaging results indicate that the human visual system is independently sensitive to information diagnostic for both detection and individuation. Behaviorally, visual recognition performance with image fragments improves with increasing amounts of task-specific information carried by face fragments for both tasks. With respect to neuroimaging, a number of regions in the ventral visual pathway were found that respond more robustly to fragments carrying higher levels of task-specific information relative to fragments carrying lower levels of information for both tasks. Region-of-interest analyses revealed functionally-defined face-selective regions, such as the right FFA and OFA, also showed sensitivity to detection-specific information. Given that the procedure used to localize these regions is a form of face detection, that is, comparing faces to objects, this may not be very surprising. However, we note that detection sensitivity was tested using *only* face fragments and these fragments covered less than a fifth of the area of a whole face. This suggests that some face parts are preferentially represented relative to others given their informativeness with regard to face detection. That the neural coding of features is sensitive to the demands of face detection has been previously noted [14] and is consistent with our present results. Here we show that such results, presumably due to detection sensitivity, are independent from individuation. Moreover, we extend such results to individuation and we conclude that the visual system responds to the constraints imposed by both tasks.

Third, the neural representation of faces appears to differentially reflect detection and individuation demands. Sensitivity to the former is revealed by the size of the neural response in a series of regions in the bilateral IOG and the right pFG while sensitivity to the latter is found both in the size of the neural response in one area of the right aIT as well as in the neural pattern in the right FFA. Overall, these findings support the idea of different types of neural representations underlying detection or individuation.

One specific point of contention regards the role of the FFA and the aIT in these two tasks. A considerable body of results from neuroimaging [7], [6], [40], [5], [4], [41] and neuropsychology [42]–[44] suggests that the FFA is involved in detection and individuation. However, at least one study [8] challenges this view and suggests the FFA may delegate other regions, in particular the aIT, to process faces at the individual level. On this account, the sensitivity of the FFA to face individuation demands, as revealed by neuroimaging results, could simply be due to the feedback received from such regions rather than because of its direct involvement in the task.

An interesting perspective on this issue comes from the study of congenital prosopagnosia, a condition characterized by profound impairment in face recognition, particularly at the individual level, in the absence of an obvious insult to the brain. A recent diffusion tensor imaging study [10] associated recognition performance in prosopagnosics with the degree of structural integrity of the right Inferior Longitudinal Fasciculus (ILF). ILF is one of the two major fiber tracts passing through the fusiform gyrus and connects the lingual and fusiform gyri with the superior, inferior and middle temporal gyri as well as the hippocampus and the parahippocampus. While, given the length of the tract, this result by itself may fail to directly involve the aIT, it could explain cortical volume alterations in the inferotemporal cortex observed in this population [45]. Interestingly, activation in face-selective areas in prosopagnosics and normal humans appears to be comparable [46], [47]–but see [44]. This pattern of results seems to suggest the aIT is important for face recognition and a partial breakdown in the communication between the fusiform gyrus and the aIT may be a plausible source of face individuation deficits.

Our results can help bridge two potentially divergent lines of evidence. In agreement with most neuroimaging studies, we find evidence for the direct involvement of the FFA in face individuation and against the hypothesis of an indirect feedback-conditioned role. At the same time, we also find evidence for the role of the aIT in individuation [8], a role also suggested by the neuropsychological literature. However, the FFA and the aIT turned out to exhibit two different types of sensitivity to individuation, one revealed by multivariate pattern analysis and the other by univariate analysis. This difference by itself does not explain, of course, why the same studies fail to involve both areas in individuation–they typically implicate only the FFA or aIT, but not both. In addition to the difference in sensitivity revealed by our two analysis methods, this discrepancy can be accounted for in several other ways. First, face-localizer tests are optimized primarily for detection, that is, they compare faces to other categories of objects, and thus can fail to involve neural structures that serve primarily face individuation. Second, given the special status conferred to a group of regions including the FFA as the ‘core system’ for face processing [3], neuroimaging research has particularly focused on these restricted brain areas and, thus, may fail to observe relevant activation in other areas, for example, the aIT. Third, and most importantly, our stimuli were face fragments selected based on their task-specific information instead of whole faces. Thus, our study is aimed at dealing in a more direct manner with individuation sensitivity than previous studies using whole face images.

Overall, we interpret our current results as supporting the involvement of the FFA primarily in detection and of the right aIT in individuation. However, individual face differences are already represented in the FFA. These differences seem to be further amplified and recoded in the right aIT insofar as they lead to different types of sensitivity to individuation-specific information. If this is the case, we expect the FFA to support individuation without the help of the aIT at least to some extent. However, if the features encoded in the FFA serve primarily face detection, individuation processing in this area is likely to be suboptimal. This motivates and explains the recruitment of a different area, the aIT, dedicated to a task critical in our everyday life, individuation.

Our current results are also interesting from the perspective of the hierarchy of visual processing along the ventral visual pathway [48]. The idea that visual features of increasing complexity build successively upon each other at different levels of visual processing has been incorporated in many neurally-inspired models of object [16], [49] and face recognition [17]. More recently, this approach has also been extended to fragment-based processing [50], [51]. Composing larger, more specialized fragments successively out of smaller and more generic fragments across a series of representational levels is a computationally attractive means for instantiating hierarchical processing. Relevant to our study, this hints that larger individuation-dedicated fragments may be separately represented and built upon smaller detection-dedicated fragments. One concrete possibility suggested by the present results is that features optimal for individuation are represented as *patterns* over face detection features within the FFA and then recoded in a more localist fashion within the right aIT. The neural plausibility of this hypothesis is the goal of further research.

Finally, our results reinforce the assumption that overlapping image fragments provide a neurally-plausible representational schema for object features. The argument the present study makes for their plausibility and utility is that they help clarify how different tasks shape the neural code underlying face recognition. However, we should also acknowledge the limits of this approach as an actual model. One question regards the effectiveness of using rectangular fragments to represent what are, most likely, non-rectangular features. In response to this, we take rectangular-shaped features to be a rough but reasonable approximation of the actual features encoded by the visual system. More plausible features with smoother edges without sharp corners should be furthered examined. However, we argue, our investigation of task-specific information as carried by fragments is systematic and sufficiently detailed that the precise shape of the features should not alter significantly the conclusions we reached above. Consistent with this, replacement of square-like features with circular ones did not significantly alter measurements of detection-specific information in a related study [14]. Another more critical issue concerns the manner in which fragments are actually represented by the neural code. For instance, every fragment contains a multitude of cues with different contributions to various recognition tasks. How such cues are separately considered, encoded and integrated into a unified representation is an important problem that should be addressed further. For present purposes, we treat rectangular image patches as reasonable stand-ins for the actual biological representations of different face parts and subparts.

To conclude, we examined and found that face detection and individuation place different task constraints on the representational code required for automatic and human face recognition. More generally, we interpret these results as further evidence for the soundness of fragment-based models of human object processing.

## Supporting Information

Figure S1 Training set of face images
(0.48 MB TIF)

Figure S2 Natural image fragments erroneously labeled as face fragments by the method
(0.08 MB TIF)
