# Supplementary figures and images for: Task-Specific Codes for Face Recognition: How they Shape the Neural Representation of Features for Detection and Individuation
Source: PLoS One. 2008 Dec 29;3(12):e3978. doi: 10.1371/journal.pone.0003978 (PMC2607027; doi:10.1371/journal.pone.0003978)

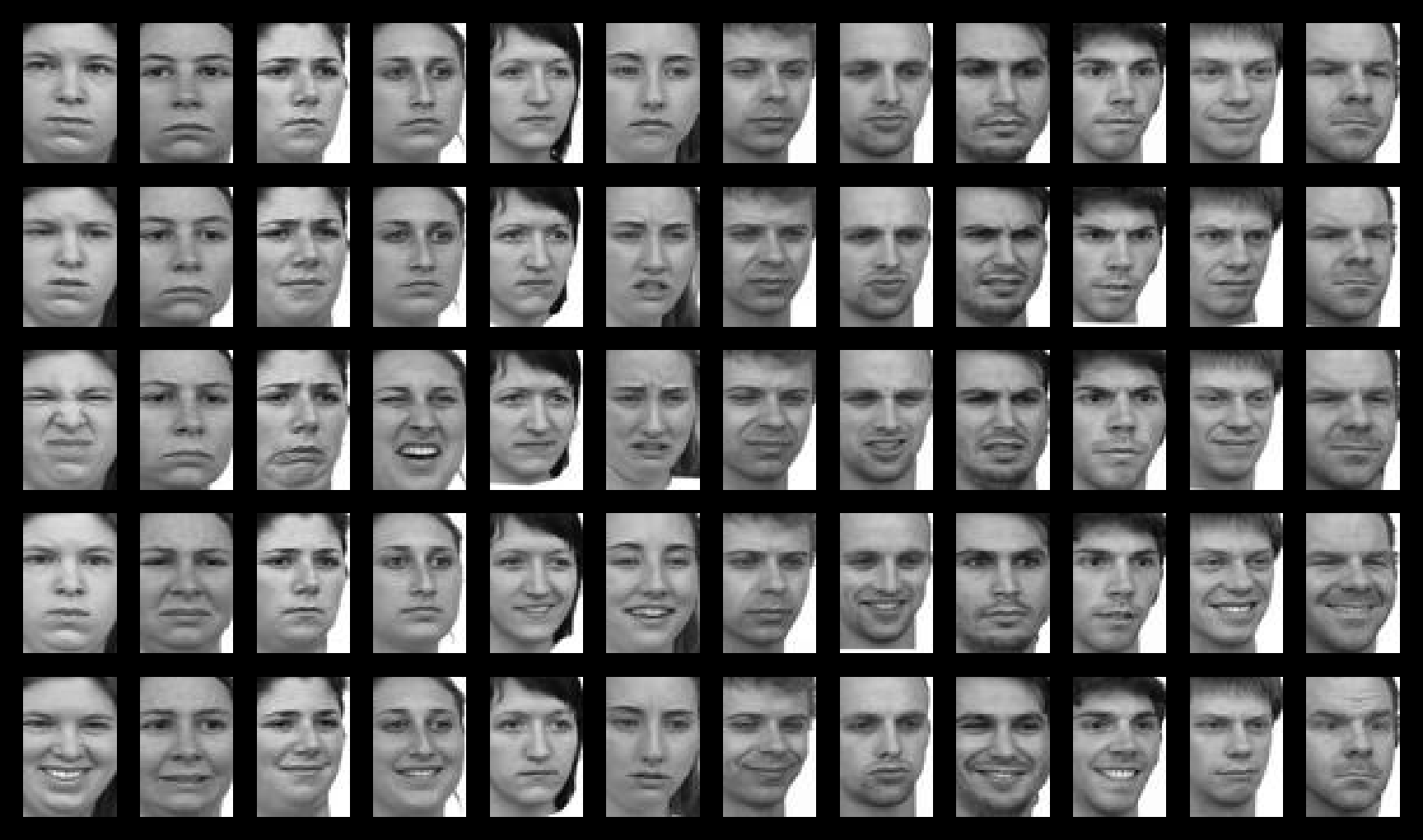

Supplement: Figure S1 — Training set of face images (0.48 MB TIF) [file pone.0003978.s001.tif]

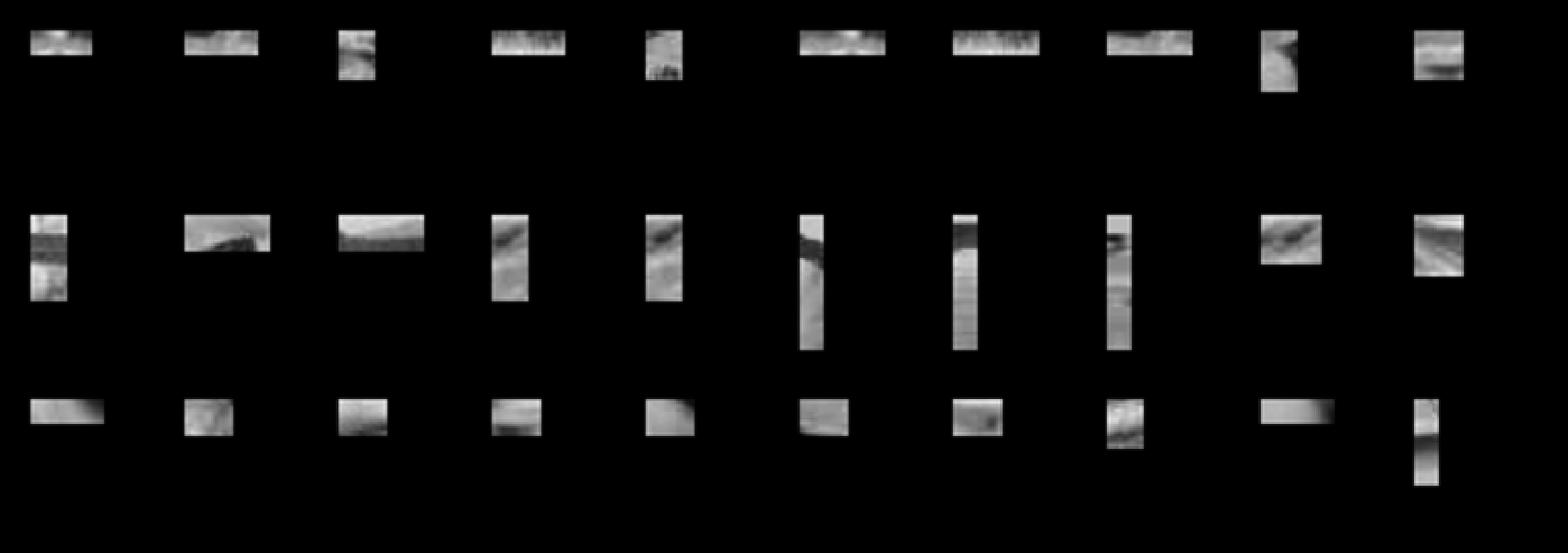

Supplement: Figure S2 — Natural image fragments erroneously labeled as face fragments by the method (0.08 MB TIF) [file pone.0003978.s002.tif]
